# Modulation of Ocular Surface Glycocalyx Barrier Function by a Galectin-3 N-terminal Deletion Mutant and Membrane-Anchored Synthetic Glycopolymers

**DOI:** 10.1371/journal.pone.0072304

**Published:** 2013-08-19

**Authors:** Jerome Mauris, Flavio Mantelli, Ashley M. Woodward, Ziyhi Cao, Carolyn R. Bertozzi, Noorjahan Panjwani, Kamil Godula, Pablo Argüeso

**Affiliations:** 1 Schepens Eye Research Institute and Massachusetts Eye and Ear, Department of Ophthalmology, Harvard Medical School, Boston, Massachusetts, United States of America; 2 Department of Ophthalmology, Center for Vision Research, Tufts University Medical School, Boston, Massachusetts, United States of America; 3 Departments of Chemistry, Molecular and Cell Biology and Howard Hughes Medical Institute, University of California, United States of America; 4 Materials Sciences Division and The Molecular Foundry, Lawrence Berkeley National Laboratory, Berkeley, California, United States of America; University of Liverpool, United States of America

## Abstract

**Background:**

Interaction of transmembrane mucins with the multivalent carbohydrate-binding protein galectin-3 is critical to maintaining the integrity of the ocular surface epithelial glycocalyx. This study aimed to determine whether disruption of galectin-3 multimerization and insertion of synthetic glycopolymers in the plasma membrane could be used to modulate glycocalyx barrier function in corneal epithelial cells.

**Methodology/Principal Findings:**

Abrogation of galectin-3 biosynthesis in multilayered cultures of human corneal epithelial cells using siRNA, and in galectin-3 null mice, resulted in significant loss of corneal barrier function, as indicated by increased permeability to the rose bengal diagnostic dye. Addition of β-lactose, a competitive carbohydrate inhibitor of galectin-3 binding activity, to the cell culture system, transiently disrupted barrier function. In these experiments, treatment with a dominant negative inhibitor of galectin-3 polymerization lacking the N-terminal domain, but not full-length galectin-3, prevented the recovery of barrier function to basal levels. As determined by fluorescence microscopy, both cellobiose- and lactose-containing glycopolymers incorporated into apical membranes of corneal epithelial cells, independently of the chain length distribution of the densely glycosylated, polymeric backbones. Membrane incorporation of cellobiose glycopolymers impaired barrier function in corneal epithelial cells, contrary to their lactose-containing counterparts, which bound to galectin-3 in pull-down assays.

**Conclusions/Significance:**

These results indicate that galectin-3 multimerization and surface recognition of lactosyl residues is required to maintain glycocalyx barrier function at the ocular surface. Transient modification of galectin-3 binding could be therapeutically used to enhance the efficiency of topical drug delivery.

## Introduction

The thick coat of carbohydrates in the glycocalyx that emerges from apical membranes of epithelial cells is critical to maintaining barrier function on mucosal surfaces. This glycocalyx is important in preventing access of microbes to plasma membranes, but also significantly restricts drug and vaccine targeting of epithelial cells [1]. In the eye, the bioavailability of topical drugs is notoriously poor, in the order of 5% or less [2], [3]. Key reasons for such low bioavailability include the short precorneal residence time of ophthalmic solutions, as well as multiple permeability barriers including the apical epithelial glycocalyx [2].

Glycocalyces on mucosal surfaces are rich in transmembrane mucins, a group of high-molecular-weight glycoproteins with long filamentous structures that extend 200–500 nm above the plasma membrane—far above other glycoconjugates [4]. Stratified human corneal and conjunctival epithelia express at least three membrane-associated mucins: MUCs 1, 4, and 16 [5]. These large molecules are characterized by the presence of heavily O-glycosylated, central tandem repeats of amino acids, with their carbohydrate component providing 50–90% of the mature glycoprotein's molecular mass [6]. The O-linked carbohydrates play an important role in maintaining glycocalyx barrier function at the ocular surface by preventing apical adhesion and infection [7], [8], [9].

A molecular mechanism by which mucin O-glycans contribute to maintaining barrier function in the cornea is through interaction with galectin-3 on the apical surface of epithelial cells [10]. Galectins are a family of mammalian β-galactoside-binding proteins that share highly conserved, carbohydrate-recognition domains (CRDs). Galectin-3 is the exclusive member of the chimera-type galectin subgroup that contains one CRD connected to an extended non-lectin N-terminal domain [11]. As determined by sedimentation velocity and equilibrium experiments, galectin-3 is predominantly monomeric in solution [12]. Moreover, it can form homodimers by self-association through its CRDs in the absence of its saccharide ligands [13]. However, in the presence of its carbohydrate-binding ligands, galectin-3 can polymerize through its N-terminal domain [13], [14], [15], [16]. Multimerization of galectin-3 often leads to cross-linking of its saccharide ligands and formation of lattice-like structures on plasma membranes essential for the biological activity of the cell [17], [18], [19].

Limited information is available on the precise organization of the glycocalyx barrier in the most apical layer of the corneal epithelium, and whether it can be transiently modified to allow targeted delivery of ophthalmic drugs. The goal of this study was to evaluate the role of the galectin-3 N-terminal polymerizing domain in the modulation of corneal epithelial glycocalyx barrier function, and to determine whether synthetic glycopolymers can be anchored to corneal epithelial plasma membranes to interfere with galectin-3 binding.

## Results

### Galectin-3 maintains corneal epithelial barrier function *in vitro* and *in vivo*

**Figure 1:**
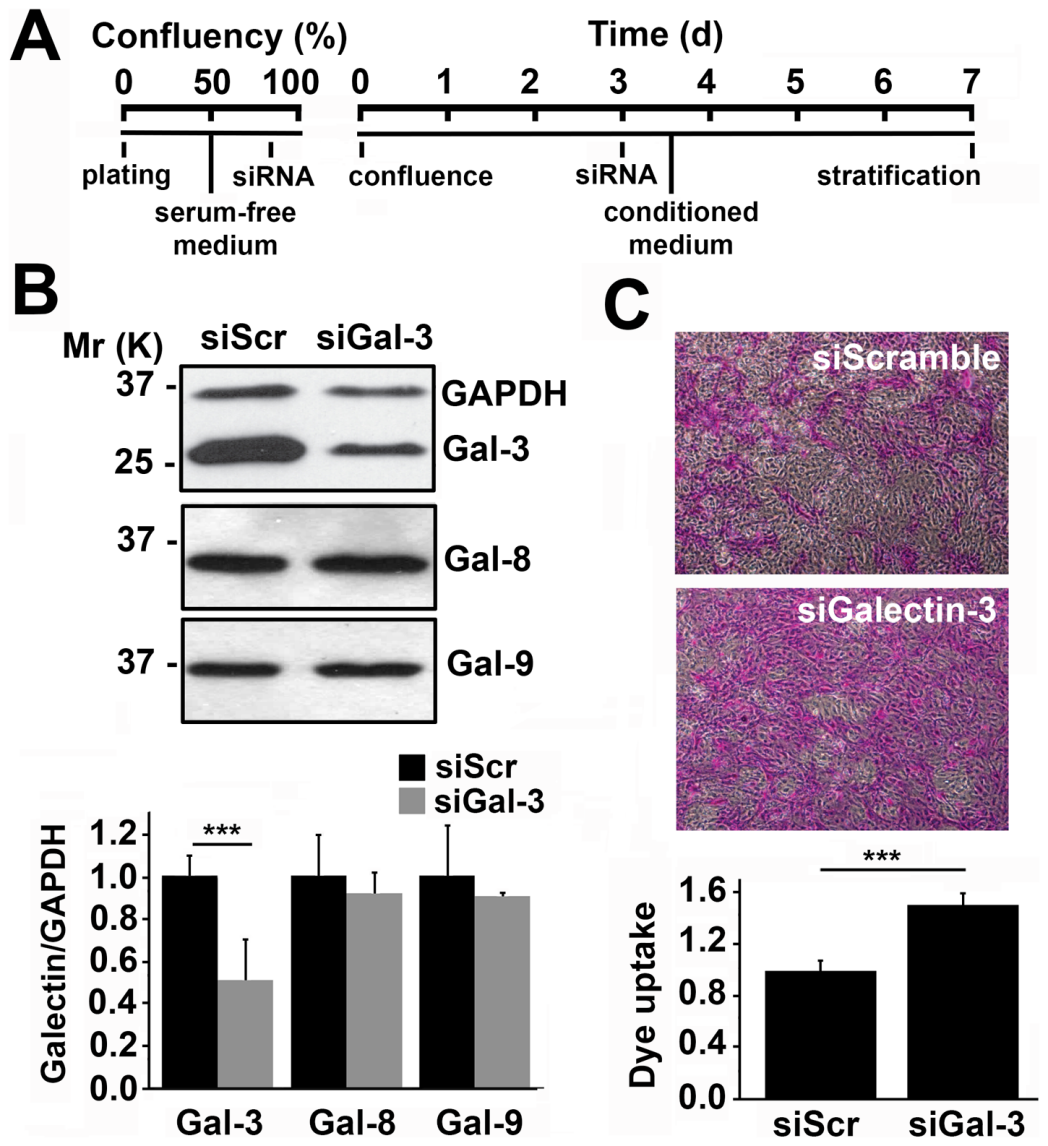
Galectin-3 maintains corneal epithelial barrier function *in vitro*. (A) Timeline illustrating the transient abrogation of galectin-3 in a three-dimensional culture system using siRNA. (B) Analyses of whole corneal epithelial cell lysates revealed a 51±18% galectin-3 protein reduction in cultures treated with galectin-3 siRNA (siGal3) as compared to scramble siRNA (siScr). Galectin-3 knockdown did not affect expression of galectin-8 and -9. The upper panel shows representative western blots. (C) The average area of rose bengal staining after galectin-3 knockdown was 51±9% higher than in scramble cells. Representative images for each condition are shown in the upper panel. Images were obtained using a 10× objective lens. All the experiments were performed at least in triplicate and represent the mean ±SD. ***P<0.001.

To address the direct contribution of endogenous galectin-3 to epithelial barrier function, galectin-3 expression was first transiently abrogated in a three-dimensional culture system with multilayered human cells using siRNA (Figure 1A). As shown by western blot, transfecting human corneal-limbal epithelial (HCLE) cells with galectin-3 siRNA twice—at 80% confluence and 3 days post-confluence—reduced galectin-3 protein levels by 51±18% compared to scramble control (Figure 1B). In these experiments, abrogation of galectin-3 did not alter the biosynthesis of either galectin-8 or -9, two additional galectins expressed by the human ocular surface epithelia [20] (Figure S1).

Next, we used the rose bengal diagnostic dye to determine the effect of galectin-3 abrogation on epithelial barrier function. In this assay, protection from rose bengal penetration into epithelial cells is indicative of a fully functional mucosal barrier, whereas penetration and positive staining of the epithelia indicates the presence of a compromised glycocalyx barrier [10]. As shown in Figure 1C, silencing of galectin-3 in human corneal epithelial cells led to a statistically significant increase in dye uptake compared to that of scramble control, indicating loss of barrier function after galectin-3 abrogation.

**Figure 2:**
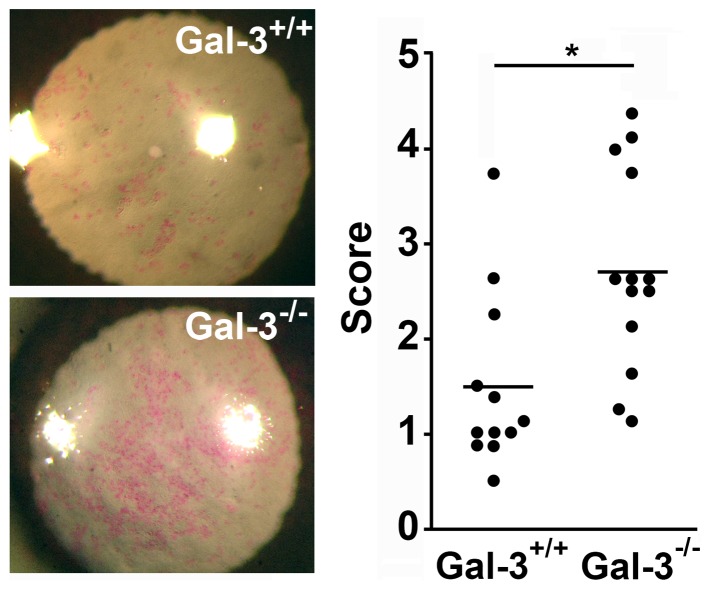
Abrogation of galectin-3 impairs barrier function in mouse corneas. Numerical scoring for the intensity of staining with rose bengal revealed a higher incidence of epithelial defects in corneas of galectin-3 null mice (Gal-3^−/−^) as compared to wild-type (Gal-3^+/+^) controls. Representative images are shown in the left panel. *P<0.05.

To further evaluate the effect of galectin-3 on barrier function *in vivo*, we examined the ocular surface of galectin-3 null mice. For these experiments, whole eye globes were surgically removed and incubated with a rose bengal solution for 60 seconds. Corneas of galectin-3 null mice were characterized by a significant increase in the number of punctate epithelial defects, corresponding to areas of superficial epithelial cells with enhanced rose bengal staining, as compared to those of wild type animals (Figure 2).

### Disruption of galectin-3 binding and multimerization impairs glycocalyx barrier function

**Figure 3:**
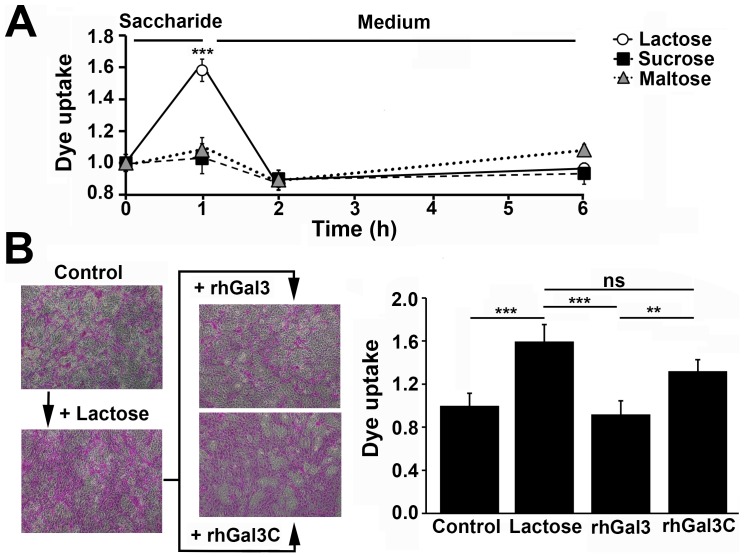
Disruption of galectin-3 binding and multimerization impairs glycocalyx barrier function. (A) One-hour preincubation of stratified human corneal epithelial cell cultures with β-lactose—but not with the non-inhibitory controls of galectin binding, sucrose and maltose—resulted in a significant and transient increase in rose bengal uptake. (B) Incubation with rhGal3 after treatment with β-lactose allowed recovery of barrier function in corneal epithelial cells. On the other hand, addition of rhGal3C resulted in sustained rose bengal uptake by the cell culture. Representative images are shown in the left panel. Images were obtained using a 10× objective lens. All the experiments were performed in triplicate and represent the mean ±SD. ns, not significant, **P<0.01, ***P<0.001.

We have previously reported that incubation of HCLE cells with competitive carbohydrate inhibitors of galectin binding (β-lactose or modified citrus pectin) impairs barrier function [10]. To gain insight into whether barrier function can be transiently disrupted, HCLE cells were preincubated with β-lactose, followed by incubation in basal medium for up to 6 hours. As previously described, incubation with β-lactose, but not with the non-inhibitory controls sucrose and maltose, resulted in a significant increase in rose bengal uptake by the stratified cultures of corneal epithelial cells (Figure 3A). Interestingly, removal of the β-lactose-containing medium from the cell culture resulted in the recovery of barrier function to basal levels within an hour, suggesting a mechanism by which the cell-surface is repopulated by galectin-3 after competitive inhibition as previously described [21].

In additional experiments, we cloned and purified full-length galectin-3 (rhGal3) and a galectin-3 mutant (rhGal3C) lacking the N-terminal polymerization domain to determine whether galectin-3 multimerization is necessary to provide glycocalyx barrier function. As expected, incubation of corneal epithelial cell cultures with rhGal3 for 1 hour at 4°C after treatment with β-lactose allowed protection against rose bengal uptake (Figure 3B). In contrast, addition of rhGal3C prevented recovery of barrier function, indicating that galectin-3 multimerization is required to maintain the integrity of the corneal epithelial glycocalyx.

### Cellobiose glycopolymers incorporate into cell membranes to modify glycocalyx integrity

**Figure 4:**
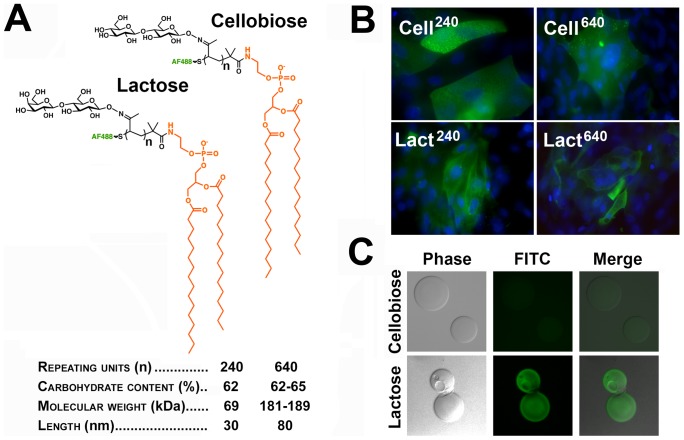
Synthetic glycopolymers incorporate into stratified cultures of human corneal epithelial cells. (A) Schematic structure and properties of Alexa Fluor 488 cellobiose- and lactose-containing glycopolymers functionalized with a phospholipid end group. (B) Fluorescence microscopy images demonstrated that, following a 1-hour incubation, glycopolymers (green) with 240 and 640 repeating units incorporated into islands of stratified corneal epithelial cells. DAPI was included in the mounting medium to localize the position of the nuclei (blue) in the cell culture. Images were obtained using a 40× objective lens. (C) By pull-down assay, synthetic glycopolymers with lactose-decorated backbones, but not cellobiose derivatives, bound to an rhGal3 affinity column.

The exogenous insertion of synthetic bioactive polymers is a recently reported alternative approach to manipulating cell surfaces in living cells [22]. Artificial mucin-like glycoconjugates mimicking their natural counterparts have been designed by adding carbohydrates to synthetically tractable polymer backbones. These large backbones are then linked to a terminal hydrophobic phospholipid tail for anchoring into lipid bilayers [23], [24]. Here, we evaluated whether fluorescent glycopolymers with a lipid anchor and chain-length distributions of 30 and 80 nm (corresponding to 240 and 640 repeating units, respectively) could be inserted into cultures of stratified human corneal epithelial cells to modify the character of the cell surface. In these experiments, two types of glycopolymers, featuring cellobiose- and lactose-decorated polymeric backbones (Figure 4A), were used to establish the relevance of lactosyl residues to barrier function.

As determined by fluorescence microscopy, cells treated with glycopolymers showed cell surface fluorescence, independently of the chain length distribution and carbohydrate content of their polymeric backbones (Figure 4B). Incorporation was dependent on the phospholipid tail, as control glycopolymers lacking this hydrophobic domain showed no significant insertion into cells (data not shown). Binding of lactose-containing glycopolymers, but not their cellobiose counterparts, to recombinant human galectin-3 was confirmed by pull-down assay (Figure 4C).

**Figure 5:**
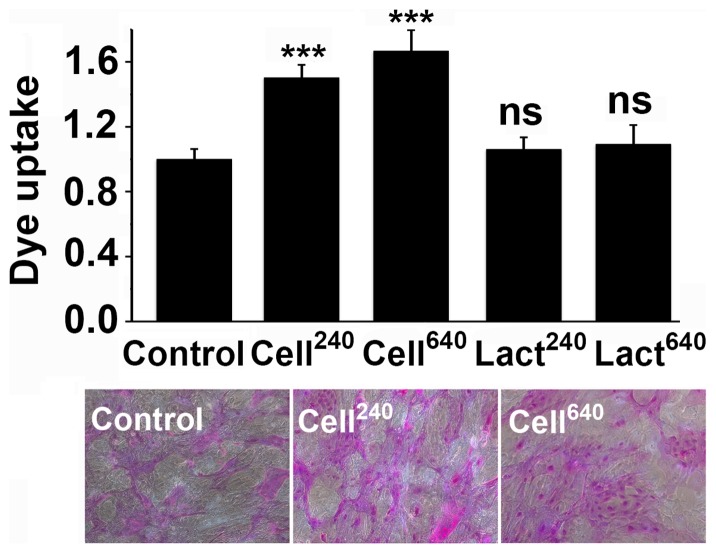
Cellobiose glycopolymers impair glycocalyx barrier function in human corneal epithelial cells. As determined using the rose bengal penetration assay, treatment of stratified corneal epithelial cells for 1 hour with cellobiose glycopolymers, containing either 240 or 640 repeating units, significantly impaired glycocalyx barrier function. No effect on rose bengal uptake was observed when lactose-containing glycopolymers were used. Images were obtained using a 10× objective lens. All the experiments were performed in triplicate and represent the mean ±SD. ns, not significant; ***P<0.001.

In subsequent experiments, we tested the effect of glycopolymer insertion on barrier function using the rose bengal penetration assay (Figure 5). Treatment of stratified corneal epithelial cells with cellobiose-containing glycopolymers led to a significant increase in rose bengal uptake, suggesting that surface insertion of cellobiose residues with no affinity for galectin-3 disrupts the glycocalyx barrier. On the other hand, treatment with lactose-containing glycopolymers had no effect on rose bengal uptake as compared to untreated cells.

## Discussion

Maintenance of an effective epithelial barrier on exposed mucosal surfaces requires both trans- and paracellular exclusion of macromolecules and microorganisms. The intercellular tight junction that connects individual epithelial cell membranes serves as the rate-limiting paracellular barrier [25]. Transmembrane mucins and their associated O-glycans, on the other hand, maintain the integrity of the epithelial glycocalyx and provide a transcellular barrier to particles and pathogens [26], [27], [28]. While functioning as a protective mechanism to exposed surfaces, this resistance to apical internalization also impairs the delivery of therapeutic formulations into mucosal surfaces. Overcoming these barriers in a transient manner is, therefore, an alternative approach to efficiently improving drug entry from topical administration.

Through studies performed during the last decade, it has become apparent that transmembrane mucins bind galectins in a carbohydrate-dependent manner to elicit a variety of biological functions under both physiological and pathological conditions [10], [29], [30]. In human corneal epithelial cells, competitive inhibition of galectin binding and abrogation of c1galt1—a critical galactosyltransferase required for the synthesis of core 1 mucin O-glycans—has been associated with loss of barrier function [10], [31]. In addition to galectin-3, several members of the galectin family, such as galectins -8 and -9 [20], [32] and galectins -1 and -7 [33], have been detected in human and mouse ocular surface epithelial cells, respectively. In our experiments, selective abrogation of galectin-3 biosynthesis *in vitro* and *in vivo* resulted in increased permeability to the rose bengal diagnostic dye (Figures 1,2), indicating a role for this galectin in maintaining glycocalyx barrier function. Interestingly, lack of galectin-3 did not lead to complete abrogation of barrier function, suggesting that other carbohydrate-binding proteins may contribute to maintaining the integrity of the epithelial glycocalyx. Evidence indicates that multiple members of the galectin family can recognize mucin-type O-glycans as biological counter-receptors on cell surfaces [30], [34], [35], supporting the possibility of a redundant function for these proteins in maintaining barrier function at the ocular surface.

In addition to its carbohydrate recognition domain, the biological function of galectin-3 is also regulated by the non-lectin N-terminus. This region not only mediates multimerization, but also shows positive cooperativity in lectin binding to immobilized ligand clusters [15], [36]. In our experiments, short-term addition of a competitive inhibitor of galectin binding resulted in transient disruption and subsequent recovery of barrier function (Figure 3). This result suggests a mechanism, previously shown in SUDHL-6 cells [21], by which the cell-surface is repopulated by galectin-3 soon after removal of the inhibitor from the cell culture. The recovery of barrier function, however, was impaired in the presence of rhGal3C, a galectin-3 mutant lacking the N-terminal domain, indicating that disruption of galectin-3 multimerization during the recovery process impairs barrier function. Interestingly, the galectin-3 N-terminal domain can be proteolytically cleaved by matrix metalloproteinases (MMPs), particularly MMP2 and MMP9 [19]. As increased levels of MMPs are commonly associated with ocular surface disease [37], [38], we speculate that proteolytic cleavage of galectin-3 under pathological conditions may contribute to the increased uptake of rose bengal and loss of barrier function commonly observed in these patients.

Identifying the factors that facilitate or hinder association between galectins and transmembrane mucins is not only critical to understanding the organization of the epithelial glycocalyx, but also may be exploited for potential therapeutic development. Synthetic glycopolymers that emulate natural mucins have been developed during the past few years to study how the structure of mucin glycans and their spatial arrangements along the mucin's polypeptide backbone affect the interactions with carbohydrate-binding proteins [39], [40]. Glycopolymers functionalized with lipid tails have been introduced into membranes of live cells such as ldlD CHO, a cell type lacking endogenous mucins [23]. Here, we show that glycopolymers decorated with pendant cellobiose- and lactose-glycans incorporate into cultures of stratified human corneal epithelial cells (Figure 4), known to contain apical islands of undifferentiated and differentiated cells, the latter featuring glycosylated transmembrane mucins [41], [42]. Increasing the amount of cellobiose on the cell surface via glycopolymer insertion enhanced rose bengal uptake (Figure 5), suggesting that interference with surface recognition of endogenous lactosyl residues impairs barrier function at the ocular surface. Unexpectedly, insertion of lactose-containing glycopolymers, which have the capacity to bind galectin-3, did not enhance barrier function in our three-dimensional culture system; in fact, the regions of rose bengal uptake detected were similar to those of control cultures. A possible explanation is that lactose-containing glycopolymers incorporate into the glycocalyx but fail to compete for galectin-3 binding in the presence of endogenous glycosylated mucins—natural ligands for galectin-3 on apical surfaces [10], [30]. Alternatively, lactose-containing glycopolymers may incorporate into undifferentiated apical cells with poorly glycosylated mucins, but in insufficient quantities to efficiently induce lattice formation. As restoring barrier function is essential to the treatment of ocular surface disease, further research is required to elucidate the underlying causes that may impair the gain of glycocalyx barrier function when synthetic glycopolymers are used.

Overall, data in this study indicate that both multimerization of galectin-3 and surface recognition of lactosyl residues are required to maintain glycocalyx barrier function at the ocular surface. Studies aiming to determine whether the ocular surface glycocalyx can be manipulated therapeutically to enhance bioavailability of topical drugs are likely to lead to greatly improved treatment for ocular surface diseases.

## Methods

### Ethics Statement

All animal procedures in this study were performed in accordance with the Association for Research in Vision and Ophthalmology Resolution on the Use of Animals in Vision Research, the recommendations of the National Institutes of Health Guide for the Care and Use of Laboratory Animals, and approved by Tufts University Division of Laboratory Animal Medicine in Boston, MA; Protocol # B2011-15. The techniques used for the development of the human corneal-limbal epithelial cell line (kindly provided by Dr. Ilene Gipson; Schepens Eye Research Institute; Boston, MA) have been previously described [41].

### Mice

Galectin-3 null (Gal3^−/−^) mice were generated by homologous recombination on a C57BL/6 background as described previously [43]. Six- to eight-week-old, Gal3^−/−^ and wild type mice (14 and 12 animals per group, respectively) were used.

### Cell culture

Telomerase-immortalized human corneal-limbal epithelial (HCLE) cells were plated at a seeding density of 5×10^4^ cells/cm^2^. HCLE cells were maintained at 37°C in 5% CO_2_ and grown in GIBCO keratinocyte serum-free medium (KFSM) supplemented with bovine pituitary extract, 0.2 ng/ml epithelium growth factor (EGF) and 0.4 mM CaCl_2_. Once confluent, cells were switched to Dulbecco's modified Eagle's medium/F-12 (DMEM/F12) supplemented with 10% calf serum and 10 ng/ml EGF for 7 days to promote cell stratification and establishment of barrier function [42].

### Cloning and purification of full-length galectin-3 and galectin-3 N-terminal deletion mutant

cDNA encoding human galectin-3 (LGALS3, Accession No. BAA22164.1) was amplified by polymerase chain reaction (PCR) using reverse transcribed mRNA extracted from HCLE cells. The amplification was performed in a 20-µl reaction volume containing 2 µl of desalted cDNA, 200 µM dNTP, 0.5 µM of 5′ and 3′ primer, and 1 unit of Phusion® high-fidelity DNA polymerase (New England Biolabs, Ipswich, MA) in 1x Phusion® HF buffer. The 5′ and 3′ primer sequences containing NdeI and BamHI cloning sites (bolded) were, respectively, 5′-GGCGGCGGCGGCTCTAGA**CATATG**GCAGACAATTTTTCGCTCCATGATGC-3′ (primer 1) and 5′-GGCGGCGGCGGC**GGATCC**GCTCTTCCGCATTATATCATGGTATATGAAGCACT-3′ (primer 2). The samples were placed in a MyCycler™ Thermal Cycler (Bio-Rad Laboratories Inc.; Hercules, CA) programmed for a temperature-step cycle of 98°C (30 seconds) and 72°C (40 seconds) for 25 cycles. After the final cycle, the reaction was maintained at 72°C for 10 minutes. The final reaction products were resolved on a 0.75% agarose gel containing ethidium bromide (0.5 µg/ml) (Figure S2A). The PCR product was cloned into a pTWIN2 vector (New England Biolabs) by restriction endonuclease digestion using NdeI and BamHI (New England Biolabs). rhGal3C, a truncated form of galectin-3 lacking the first 62 amino acids in the N-terminal polymerizing domain, was generated from the pHGal3 plasmid using the Phusion™ Site-Directed Mutagenesis Kit (New England Biolabs) (Figure S2B). The 5′ and 3′ phosphorylated primers were, respectively, 5′Phos-TACCCTGGAGCACCTGG-3′ (primer 3) and 5′Phos-CATATGTATATCTCCTTCTTAAAGTTAAACA-3′ (primer 4). The sample was placed in a MyCycler™programmed for a temperature-step cycle of 98°C (30 seconds) and 72°C (2 minutes) for 25 cycles. After the final cycle, the reaction was maintained at 72°C for 10 minutes. The PCR product was resolved in an agarose gel for size verification and DNA quantification, and then ligated. Plasmids were sequenced at the DNA Core Facility, Massachusetts General Hospital, Boston, MA.

Both expression constructs were transformed into *E. coli* Rosetta™ cells (Novagen, Madison, WI). Positive transformants were selected in agar plates and grown at 37°C with shaking in LB medium (10 g/l tryptone, 5 g/l yeast extract, 10 g/l NaCl, 1 g/l dextrose, and 1 g/l MgCl_2_, pH 7.2) supplemented with ampicillin (100 µg/mL) and chloramphenicol (34 µg/ml) to an OD_600_ of 0.5–0.8. Heterologous protein expression was induced by the addition of 0.3 mM IPTG (American Bioanalytical, Inc.; Natick, MA), and the induced cultures incubated at 15°C overnight with shaking. Bacterial cultures were then centrifuged at 10,000×g for 10 minutes at 4°C, and the supernatant discarded. Bacterial pellets were resuspended in lysis buffer (20 mM Tris, pH 8.0, 5 mM EDTA, 10 mM sucrose, 20 mM β-mercaptoethanol) and sonicated at 4°C, over three 60-second cycles, separated by 1-minute intervals. Lysates were then clarified at 10,000×g for 20 minutes and used immediately.

rhGal3 and rhGal3 C were purified from lysates by affinity chromatography using lactosyl sepharose as described previously [44]. Protein content in elution fractions was determined using the BCA Protein Assay Kit (Pierce; Rockford, lL). Aliquots (10 µl) were run on a 10% SDS-PAGE gel and analyzed by GelCode® Blue Stain (Thermo Fisher Scientific; Rockford, IL) to assess the purity of the protein preparation. Fractions enriched in recombinant protein were pooled, and the identity of the purified recombinant protein further confirmed by immunoblot (Figure S2) as described below. To eliminate contaminating bacterial endotoxins, rhGal3 and rhGal3 C were further purified by polymyxinB affinity chromatography (Sigma-Aldrich; St. Louis MO). The absence of lipopolysaccharide was confirmed using ToxinSensor**™** Chromogenic LAL Endotoxin Assay Kit (GenScript; Piscataway, NJ) following the manufacturer's instructions. Protein solutions were concentrated by filtration using a Vivaspin 20 centrifugal concentrator (10 kDa molecular weight cut-off; GE Healthcare; Littleton, MA), dialyzed against PBS buffer containing 10% of glycerol, and stored at −20°C.

### Galectin-3 siRNA transfection

Galectin-3 was depleted using Silencer® Select Pre-designed siRNA (S8149; Ambion, Austin; TX, USA) targeting human LGALS3 mRNA. HCLE cells were transfected in 6-well culture plates twice, once at 80% confluence and then 3 days post-confluence, with galectin-3 siRNA or scramble control. For each transfection, cells were treated with 500 nM siRNA in Opti-MEM® reduced-serum medium GlutaMAX™ (Invitrogen; Carlsbad, CA, USA) containing 1 µl/100 mm^2^ Lipofectamine™ 2000 (Invitrogen) for 6 hours. Cultures were then incubated for 20 hours with either KSFM, for cells treated at 80% confluence, or DMEM, for stratifying cells. After the final transfection, the media was switched to DMEM/F-12 to promote stratification and differentiation.

### Glycopolymer synthesis

Synthetic glycopolymers were prepared by condensation of β–aminooxylactose or β–aminooxycellobiose to poly(methylvinyl ketone) backbones monofunctionalized with Alexa Fluor 488 and endowed with a phospholipid tail for anchoring to cell membranes. The synthesis of the polymer backbone precursors with narrow chain-length distributions has been previously described in detail [45]. The synthetic glycopolymers accommodate extended conformations and insert into lipid bilayers, where they are fluid and project away from the surface [45]. The aminooxy-glycans were prepared according to published procedures [46].

The glycopolymers used in this study were prepared according to the following general procedure: AF488-labeled poly(methylvinyl ketone) backbones (1.0 mg, 0.014 mmol of keto groups) were dissolved in tetrahydrofurane (95 µl) and transferred into a 4 ml glass vial containing a solution of β-aminooxy-glycan (6.1 mg, 0.017 mmol, 1.2 equiv per ketone group) in sodium acetate buffer (95 µl, 100 mM, pH = 5.2). The vials were placed in a heating block set at 50°C and heated for 12 hours. Next, the solvents were removed and additional acetate buffer (100 µl) was added. The tubes were heated at 50°C for an additional 18 hours. The crude reaction mixtures were then loaded onto a Sephadex G-25 (PD-10) desalting column. The polymers were eluted with DI water, and the collected fractions were lyophilized to give orange glycopolymers in >90% isolated yield. Based on ^1^H NMR analysis, approximately 62–65% of the pendant keto groups in the resulting glycopolymers were conjugated with a glycan. ^1^H NMR spectra of all polymers were collected in D_2_O on a Bruker Biospin Advance II, 500 MHz, High Performance NMR spectrometer with multinuclear CP-MAS probe and results are included in Supporting Information (Figure S3). Specifications for individual glycopolymers are as follows:

Cell^240^: repeating units, n,  = 240, cellobiose content  = 62% n, Mw = 69 kDa, isolated yield  = 3.7 mg (100%), estimated length, l, ∼30 nm.
Cell^640^: repeating units, n,  = 640, cellobiose content  = 65% n, Mw = 189 kDa, isolated yield  = 3.8 mg (93%), estimated length, l, ∼80 nm.
Lac^240^: repeating units, n,  = 240, lactose content  = 62% n, Mw = 69 kDa, isolated yield  = 3.7 mg (100%), estimated length, l, ∼30 nm.
Lac^640^: repeating units, n,  = 640, lactose content  = 62% n, Mw = 181 kDa isolated yield  = 4.0 mg (100%), estimated length, l, ∼80 nm.

For HCLE cell labeling, glycopolymers were dissolved in PBS. Serum-starved, stratified corneal epithelial cells were incubated with 2 µM glycopolymers for 1 hour at room temperature.

### Rose bengal uptake

Barrier function in cell culture was assayed by a 5-minute incubation with 0.1% rose bengal dye (Acros Organics; Morris Plains, New Jersey) as described previously [47]. For rose bengal *in vitro* assay, HCLE cells were serum-starved for 2 hours, then treated with serum-free DMEM/F12 medium supplemented with 0.1 M disaccharides (β-lactose, sucrose or maltose), rhGal3 or rhGal3C (100 µg/ml), and lactose- or cellobiose-containing glycopolymers (2 µM). The extent of dye penetrance in cell culture was assessed using an inverted microscope (Nikon Eclipse TS100). Pictures were taken at 10× with a SPOT Insight Fire Wire Camera (Diagnostic Instruments, Inc.; Sterling Heights, MI). Images were processed further for dye penetrance quantification using ImageJ software (NIH, Bethesda, MD). Uptake is represented as the integrated density of stained areas, and is normalized to control conditions.

For barrier function analysis *in vivo*, mice were euthanized and whole eye globes removed surgically, as described previously [48], by an ophthalmologist who was masked to the type of mice. Enucleation was performed by gently holding the eyeball on its side with forceps, without touching the cornea, and cutting the optic nerve with curved scissors at a distance of about 2 mm from the eyeball. The whole globes were then pinned through the sclera to a culture dish using Minutien pins, and immediately embedded in PBS, pH 7.4, containing 0.1% rose bengal. The corneas were facing up during the entire procedure and care was taken to avoid any contact with them. After 60 seconds, the corneas were washed with PBS using a 1 ml plastic disposable pipette. Immediately thereafter, a second observer obtained digital images of the corneas by placing the eyes under a stereomicroscope with double gooseneck light sources and equipped with a 12 megapixel digital camera. The masked digital images were then sent in a random fashion to four independent observers who were instructed to score rose bengal corneal staining from 0 (none) to 5 (entire corneal surface) based on the surface area stained. After the results of the staining scores were provided, the blinding codes were broken, and results were matched to the type of mice, to perform statistical analysis.

### Immunoblotting

HCLE protein extracts in RIPA buffer were electrophoresed on 10% SDS-PAGE gels and transferred onto nitrocellulose membranes (Biorad; Hercules, CA). Membranes were then blocked with 5% Blotto (Biorad) in TBST for 1 hour at room temperature, followed by incubation overnight at 4°C with the following primary antibodies: anti-galectin-3 (H160; 1∶3,000; Santa Cruz Biotechnology; Santa Cruz, CA), anti-galectin-8 (D-18; 1∶3,000; Santa Cruz Biotechnology), anti-galectin-9 (C-20; 1∶3,000; Santa Cruz Biotechnology) and anti-GAPDH. Following incubation with the corresponding peroxidase-conjugated secondary antibody (1∶5,000; Santa Cruz), positive binding was visualized with chemiluminescence (SuperSignal West Pico substrate; Thermo Scientific) on HyBlot CL autoradiography film (Denville Scientific; Inc., Metuchen, NJ). Immunoblots were quantified using ImageJ® software (National Institutes of Health; Bethesda, MD).

### Galectin-3 affinity chromatography

An rhGal3 affinity column was prepared by coupling 5 mg of rhGal3 to cyanogen bromide-activated Sepharose 4B (GE Healthcare; Piscataway, NJ) according to manufacturer's instructions. Binding activity of rhGal3 conjugated to beads was assessed by pull-down of asialofetuin (Sigma-Aldrich) [49]. Fifty microliters of rhGal3 beads were incubated with 200 µg of asialofetuin with or without 0.1 M β-lactose, for 1 hour, at room temperature. Beads were washed 5 times with PBS before addition of SDS-PAGE sample buffer. After boiling for 5 minutes at 90°C, beads were centrifuged, and supernatant run on a 10% SDS-PAGE gel. Protein was analyzed by GelCode® Blue Stain.

For the pull-down assay, a 1 µl solution of 200 µM glycopolymers in PBS were incubated with 100 µl rhGal3-conjugated agarose beads for 1 hour at room temperature. Beads were washed 3 times with PBS to remove unbound glycopolymers and mounted on glass slides.

### Fluorescence microscopy

HCLE cells grown on culture chamber slides (Lab-Tek; Naperville, IL, USA) were rinsed in PBS and fixed in methanol at −20°C for 5 minutes. Slides were washed multiple times with PBS, mounted using Vectashield mounting medium with DAPI (Vector Laboratories; Burlingame, CA), and photographed using a fluorescence microscope (Nikon Eclipse E-800; Tokyo, Japan). Beads were mounted using Vectashield medium (Vector Laboratories) and covered with coverslips.

## Supporting Information

Figure S1 **Galectin expression at the human ocular surface.** As shown by glycogene microarray analysis, galectin-3 is the most predominant galectin detected in impression cytology samples of human conjunctival epithelium (detailed data on glycogene expression can be found at http://www.functionalglycomics.org/glycomics/publicdata/microarray.jsp; Accession # MAEXP_272_042605).
(TIF)

Figure S2 **Cloning strategy for the generation of full-length galectin-3 and a galectin-3 N-terminal deletion mutant.** (A) Galectin-3 mRNA extracted from HCLE cells was reverse transcribed, amplified by PCR, and cloned into a pTWIN2 vector using NdeI and BamHI. A 0.8-kb PCR product corresponding to full-length galectin-3 was detected by agarose-gel electrophoresis. The pHGal3 plasmid was transformed into *E. coli* Rosetta™ cells and the protein lysates purified by affinity chromatography. (B) A truncated form of galectin-3 lacking the first 62 amino acids in the N-terminal domain was obtained by site-directed mutagenesis. The identity of the purified recombinant proteins was confirmed by immunoblot. CRD, carbohydrate recognition domain; PGR, proline, glycine, and tyrosine-rich domain.
(TIF)

Figure S3 **^1^H NMR spectra of synthetic glycopolymers used in this study.**
TIF)
