# Supplementary figures and images for: Modulation of Ocular Surface Glycocalyx Barrier Function by a Galectin-3 N-terminal Deletion Mutant and Membrane-Anchored Synthetic Glycopolymers
Source: PLoS One. 2013 Aug 19;8(8):e72304. doi: 10.1371/journal.pone.0072304 (PMC3747151; doi:10.1371/journal.pone.0072304)

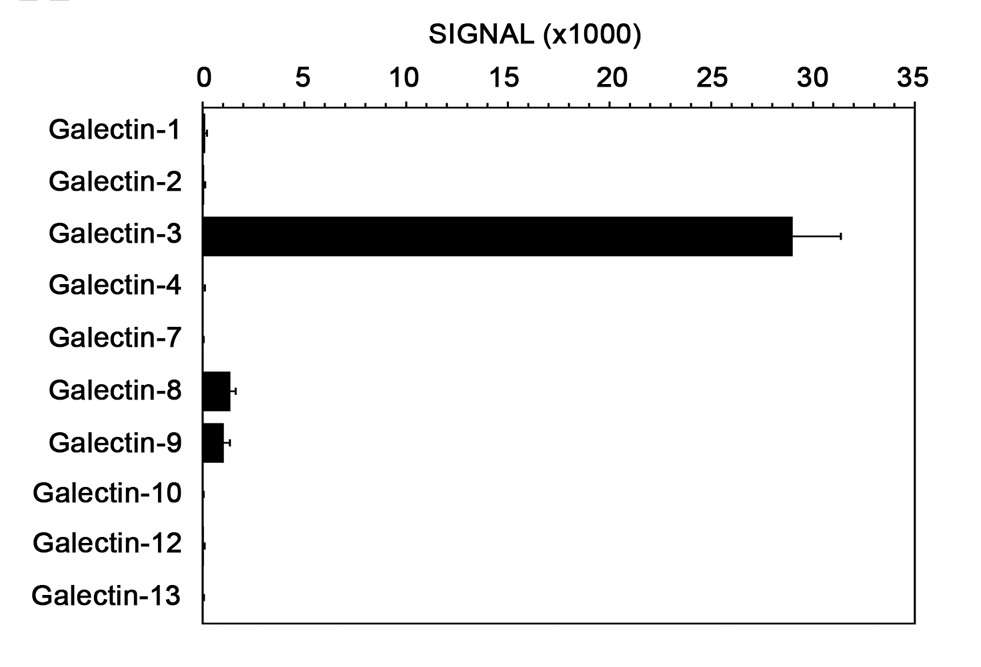

Supplement: Figure S1 — Galectin expression at the human ocular surface. As shown by glycogene microarray analysis, galectin-3 is the most predominant galectin detected in impression cytology samples of human conjunctival epithelium (detailed data on glycogene expression can be found at http://www.functionalglycomics.org/glycomics/publicdata/microarray.jsp; Accession # MAEXP_272_042605). (TIF) [file pone.0072304.s001.tif]

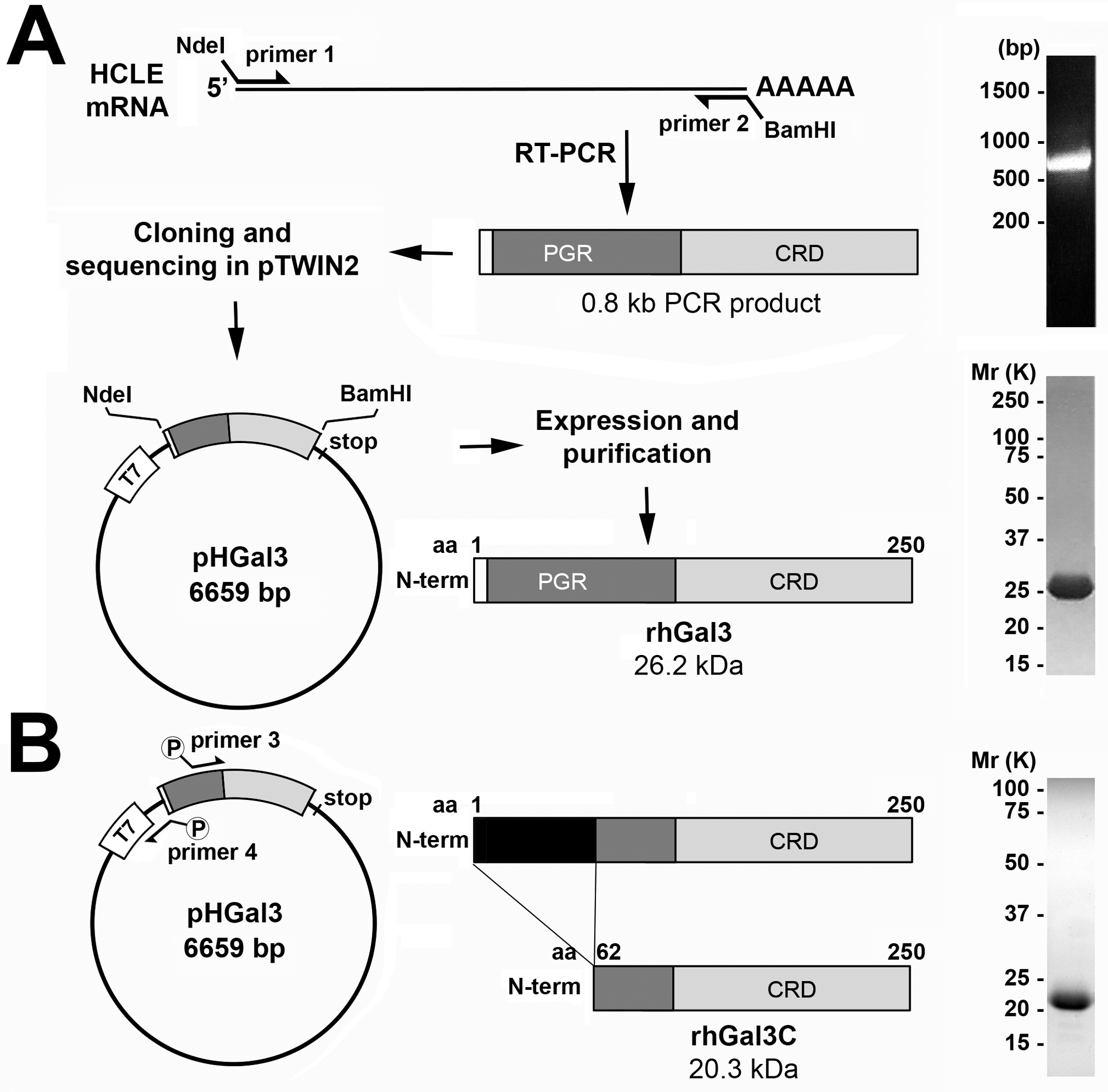

Supplement: Figure S2 — Cloning strategy for the generation of full-length galectin-3 and a galectin-3 N-terminal deletion mutant. (A) Galectin-3 mRNA extracted from HCLE cells was reverse transcribed, amplified by PCR, and cloned into a pTWIN2 vector using NdeI and BamHI. A 0.8-kb PCR product corresponding to full-length galectin-3 was detected by agarose-gel electrophoresis. The pHGal3 plasmid was transformed into E. coli Rosetta™ cells and the protein lysates purified by affinity chromatography. (B) A truncated form of galectin-3 lacking the first 62 amino acids in the N-terminal domain was obtained by site-directed mutagenesis. The identity of the purified recombinant proteins was confirmed by immunoblot. CRD, carbohydrate recognition domain; PGR, proline, glycine, and tyrosine-rich domain. (TIF) [file pone.0072304.s002.tif]

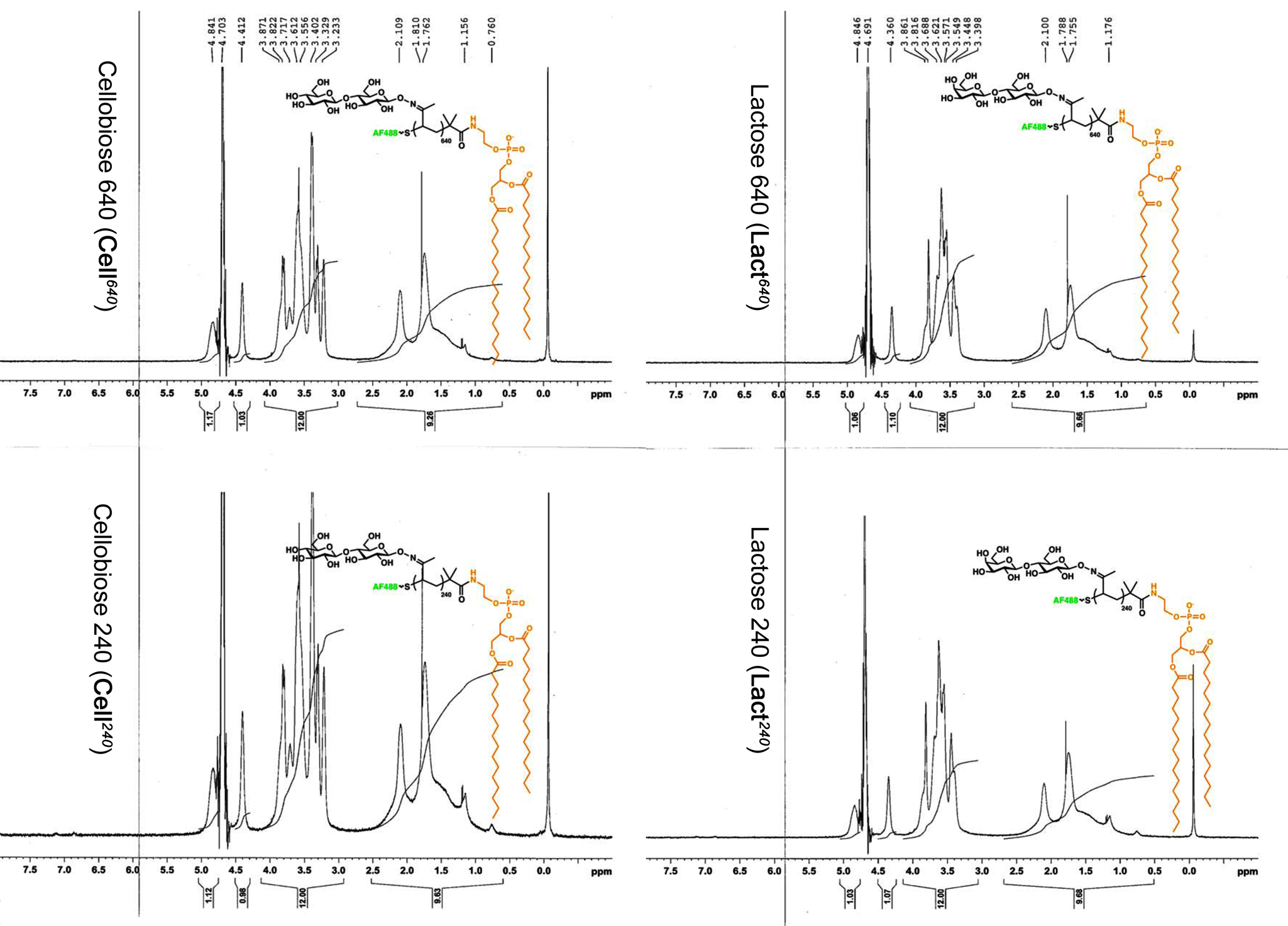

Supplement: Figure S3 — 1H NMR spectra of synthetic glycopolymers used in this study. TIF) [file pone.0072304.s003.tif]
